# Effects of Adding Tricalcium Silicate Nanoparticles to the Universal G2 Bond Adhesive as Self‐Etch Mode on the Shear Bond Strength to the Orthodontic Bracket

**DOI:** 10.1002/cre2.948

**Published:** 2024-10-25

**Authors:** Yasir R. Al‐Labban, Mehdi Alrubayee, Syed Jaffar Abbas Zaidi, Shakeel Kazmi

**Affiliations:** ^1^ Department of Orthodontics, College of Dentistry University of Baghdad Baghdad Iraq; ^2^ Department of Oral Biology, Dow Dental College Dow University of Health Sciences Karachi Pakistan; ^3^ Department of Oral Biology Shaheed Zulfiqar Ali Bhutto Medical University Islamabad Pakistan

**Keywords:** shear bond strength, tricalcium silicate, universal G2 bond

## Abstract

**Objective:**

This study investigated the effects of adding tricalcium silicate nanoparticles (TCSNp) to the universal G2 bond adhesive (G2BU) in self‐etch (SE) mode on shear bond strength (SBS) to orthodontic brackets, cytotoxicity, and degree of conversion (DC).

**Material and Methods:**

A total of 176 human teeth were divided into four groups based on TCSNp concentration in G2BU adhesive: 0% (control), 1%, 3%, and 5%. The G2BU adhesive consists of a hydrophilic primer (P) and a hydrophobic bonding agent (2B). TCSNp were added to the 2B component by mixing 0.1, 0.3, and 0.5 g of TCSNp with 9.9, 9.7, and 9.5 g of 2B, respectively. SBS was assessed after 24 h of water storage and 5000 thermocycles using a universal testing machine. Cytotoxicity was evaluated using the MTT assay on rat embryo fibroblast cells, and DC was measured using fourier‐transform infrared spectroscopy. Statistical analysis included one‐way ANOVA and Tukey's post‐hoc test, with significance set at *p* < 0.05.

**Results:**

After 24 h, mean SBS values were 15.58 MPa (control), 13.66 MPa (1% TCSNp), 15.99 MPa (3% TCSNp), and 12.04 MPa (5% TCSNp). After 5000 thermocycles, SBS values decreased to 12.91 MPa (control), 12.42 MPa (1% TCSNp), 11.11 MPa (3% TCSNp), and 10.21 MPa (5% TCSNp). ANOVA showed significant differences between groups (*p* < 0.05), except between the control and 3% TCSNp groups. Cell viability increased with higher TCSNp concentrations, with significant differences at 72 h between control and 5% TCSNp groups (*p* = 0.014). Mean DC values were 51.66% (control), 49.33% (1% TCSNp), 49.66% (3% TCSNp), and 48% (5% TCSNp). ANOVA indicated no significant differences between groups.

**Conclusions:**

Adding TCSNp to G2BU in SE mode maintains clinically acceptable SBS levels and enhances cytocompatibility. Higher TCSNp concentrations may reduce SBS and DC slightly. Further studies are needed to evaluate long‐term effects.

## 1 Introduction

Adhesive systems in dentistry can be divided into two main categories according to the etching procedure: the etch and rinse group (ER) includes two or three application steps, while the self‐etch group (SE) includes one or two application steps. In the last decade, particularly after the patent expiration for the 10 MDP molecules from Kuraray Co., the dental manufacturers introduced a new type of adhesive called multi‐mode or universal. This novel group of adhesives can be used with an acid etch technique for an ER mode or without acid etching for an SE mode. Due to this versatility, clinicians are able to choose the appropriate bonding strategy for each clinical circumstance (Sezinando 2014; Giannini et al. 2015; Ayar 2020).

Regrettably, when applied to enamel in a SE mode, universal adhesives yield inferior bond strengths compared to ER adhesives. This is a well‐documented challenge attributed to the presence of a weak acid in the universal adhesive resin. This weak acid is inadequate for sufficiently dissolving the outer enamel layer to create ample micro‐retentive porosity. Consequently, the ability of the resin to infiltrate these micro‐porosities and form resin tags is compromised (Muñoz et al. 2019).

When employed in a SE mode for bonding orthodontic brackets, the property of reduced bond strength in universal adhesives is not necessarily disadvantageous. In this context, the adhesive becomes more compatible with enamel. It is important to note that excessively high bond strength is not desirable, as debonding the orthodontic bracket at the end of fixed orthodontic treatment could result in enamel damage, such as microcracks, chipping, or even fractures. Clinically acceptable bond strength ranges between 5.9 and 7.8 MPa, and the likelihood of enamel damage escalates when bond strengths exceed 12 MPa (Reynolds 1975).

Although the SE mode may be more favorable than the ER mode, both methods present difficulties in mitigating enamel demineralization around orthodontic brackets. To address this problem, researchers have incorporated therapeutic additives such as calcium phosphate, hydroxyapatite, and fluoride into adhesive systems to deter demineralization and promote remineralization (Philip 2019; Grohe and Mittler 2021; Nassif and El Askary 2019). Furthermore, research has been conducted on including antibacterial nanoparticles such as silver, zinc oxide, and titanium dioxide (Hailan 2019). These additives are combined with the pre‐existing components of the single‐step SE primer resin, which is a complex amalgamation of organic acid and hydrophobic and hydrophilic resins. However, these additives can potentially interfere with or compromise the efficacy and bond strength of the SE system (Sezinando 2014; Garma and Ibrahim 2023). Consequently, the interactions between any therapeutic additives and the SE system must be thoroughly evaluated.

One of the additives of interest is tricalcium silicate (TCS). Due to its bioactivity, biomimetics and biocompatibility, TCS has been widely used in dentistry. It is particularly useful for endodontic and pulp treatment. The properties of TCS include its ability to adhere to the tooth structure and resin polymer with zero microleakage, bacteriostatic activity, and self‐setting behavior in a moist environment. It also releases mineral ions and induces hydroxyapatite formation (Zhao and Chang 2004; Strassler and Levin 2012; Zaidi 2021; Kaur 2017; Tan et al. 2020). Furthermore, its application in adhesives is revolutionized by its ability to significantly improve bond strength, ensuring a more resilient and long‐lasting adherence to the enamel. The biocompatible nature of TCS also ensures that it is safe for use in the oral environment, mitigating concerns about adverse reactions. Additionally, TCS's resistance to demineralization offers a protective shield for enamel against acid attacks and decay, further underscoring its importance in dental applications. This innovative use of TCS in dental adhesives marks a promising step forward in dental care and treatment.

Limited data exists on the incorporation of preventive or therapeutic nanoparticles into adhesive systems. This is especially the case for introducing TCSNp into the G2BU adhesive system (GC, Japan) when utilized in a self‐etch (SE) mode. G2BU is a novel universal two‐step adhesive about which limited information is available (Brkanović et al. 2023). The G2BU bonding mechanism is based on dual hydrophilic and hydrophobic (double H). It is supplied in two separate components: a hydrophilic primer (P) and a hydrophobic bonding agent (2B) (Brkanović et al. 2023). The partitioning of these components allows for the addition of TCSNp specifically to hydrophobic 2B during the second step. Consequently, incorporating TCSNp is unlikely to disrupt the function of the hydrophilic primer (P) or interact adversely with the hydrophobic base resin in the 2B component (Bueno et al. 2016; Gomes‐Filho et al. 2010).

Orthodontic adhesives are critical in attaching orthodontic brackets to tooth enamel. Achieving an ideal balance in shear bond strength (SBS), cytotoxicity, and the degree of conversion (DC) is essential for effective treatment and enamel safety. The recent introduction of G2 bond universal (G2BU) has presented an opportunity to improve upon existing adhesive systems, particularly with the integration of tricalcium silicate nanoparticles (TCSNp). Although TCSNp is hypothesized to offer benefits such as increased bond strength and reduced cytotoxicity, the literature currently lacks comprehensive research on the modification of G2BU with TCSNp. Specifically, there is minimal understanding of how such modification affects SBS, cytotoxicity, and DC in real‐world clinical scenarios.

While general guidelines exist for the desired range of SBS in orthodontic adhesives, there is insufficient data to confirm if the modification of G2BU with TCSNp meets or exceeds these guidelines. Previous studies have explored the cytotoxic effects of various dental adhesives, but there is a lack of research focusing on the cytotoxicity of G2BU when modified with TCSNp. Understanding cytotoxicity is crucial for assessing the material's biological safety. DC is an important factor for determining the mechanical and esthetic properties of dental adhesives, yet the literature is scant on how TCSNp modification affects the DC of G2BU. Furthermore, the efficacy of a modified application method, such as replacing enamel bur cut with pumice polishing, has not been systematically studied for G2BU, particularly when it is modified with TCSNp. Addressing these gaps is crucial for validating the clinical effectiveness and safety of modified G2BU adhesives. It will also contribute to a broader understanding of how nanoparticle integration can affect the mechanical and biological properties of orthodontic adhesives.

The objectives of this study were to evaluate the effects of adding TCSNp to the universal G2 bond adhesive in self‐etch (SE) mode on several key factors. First, the study aimed to determine the SBS of orthodontic brackets when using the modified adhesive. Second, it sought to assess the cytotoxicity of the adhesive with varying concentrations of TCSNp on rat embryo fibroblast cell lines. Third, the study aimed to measure the DC of the adhesive with different TCSNp concentrations using Fourier‐transform infrared (FTIR) spectroscopy. Finally, the study aimed to investigate the long‐term stability and durability of the adhesive bond through aging processes such as thermocycling.

## 2 Materials and Methods

### 2.1 Sample

After ethical approval from relevant ethics committee, sound extracted maxillary premolar teeth were collected. The sample size was calculated based on previous work by Munoz et al. (Takamizawa et al. 2021). using G power. The power value was Beta=80, and the alpha level was set at 5%. The sample size was calculated to be 176 human teeth, randomly distributed into four groups with 44 teeth each.

Inclusion criteria for human teeth selection included orthodontic patients aged 12–25 years with no history of surface treatment (including bleaching) who had their teeth extracted for orthodontic reasons. To confirm the absence of caries, cracks, surface defects or irregularities on the buccal enamel, a 10‐power magnification must be used (Garma and Ibrahim 2023).

Based on ISO/TS 11,405: 2015, the teeth were cleaned under tap water with a scalpel and then stored in a 1% chloramine‐T trihydrate solution for 1 week. Subsequently, the teeth were stored in distilled water until the bonding time (Gomes‐Filho et al. 2010).

This research included two phases. First, the materials were prepared by mixing the 2B component of G2BU with TCSNp. By weight, the percentage of TCSNp in the G2BU‐TCSNp mixture was: 0% (the TCSNp‐free control group), 1% (1%2B), 3% (3%2B) and 5% (5%2B). In the second phase, the adhesives were compared in terms of cytotoxicity, DC, and SBS.

### 2.2 Phase One

#### 2.2.1 Preparation of the Adhesives

The G2BU adhesive system (GC, Japan) includes two bottles: a hydrophilic primer (P) and a hydrophobic 2B applied in the second step. Subsequently to the above considerations, TCSNp (Nanoshel Co, India), which features a particle size range of 70–80 nm with a purity level of 99%, was incorporated. The following equation was used to calculate the masses of TCSNp and 2B required to achieve a preparation of 2B with 1% TCSNp.

1 g/100 g = mass of TCSNp/(mass of 2B + mass of TCSNp)/10 to obtain a small amount mass of TCSNp = 0.1 g. Mass of 2B = 9.9 g.

0.1 g + 9.9 g = 10 g = mass of TCSNp + mass of 2B.

The same equation was used for 3%2B and 5%2B (Kavrik and Kucukyilmaz 2019). The masses were obtained using a digital electronic balance with an accuracy of 0.0001 g (Precisa, Kern, USA). TCSNp was added to the 2B bottle in a black container. Four stainless‐steel balls that were 4 mm in diameter (Hartford Technology, USA) were added to aid mixing in a shaker. The electrical shaker (SYG300, China) operated at a constant shaking rate for 20 s (Dorminey, Dunn, and Taloumis 2003) in a dark room environment (Al Tuma and Yassir 2023).

### 2.3 Phase Two

#### 2.3.1 Disk Preparation and Cytotoxicity Test

For the cytotoxicity test, the sample size calculation was not applied since each type of disk was prepared from the same resin (Lee et al. 2017). For the DC sample size, the same above setting of G power was used for a previous study performed by Mohammed and Raid 2019 (Mohammed and Riad 2019); the resultant sample size was 3 for each group, and the total sample size was 12.

Thirty‐six resin disks (2 mm in height, 5 mm diameter, and 0.7065 cm^2^ in surface area) were prepared using a Teflon mold (ISO 10993‐5:2009) (Lee et al. 2017; Wawrzynkiewicz et al. 2020; Pupo et al. 2017). The polymerization was conducted using a light cure device (LED Curing Radii, SDI, Bayswater, Australia). Light intensity and time were set at 1200 mW/cm^2^ and 10 s, respectively, according to the manufacturer's instructions.

The resin was loaded into the mold and covered with a translucent polyethylene film to prevent the development of an oxygen‐inhibiting layer. The disk's thickness and diameter were measured using a digital caliper, and a special clamp ensured a standardized 2 mm distance between the light source and the specimen.

After curing, the prepared 36 disks were polished with # 1500‐grit silicon carbide paper for 15 s. The disks were sterilized using an ethylene oxide gas treatment. Next, each disk was soaked inside a sterilized glass test tube in a 1.41 mL mixture composed from Roswell Park Memorial Institute medium (RPMI 1640, Grand Island, NY, USA) containing 10% fetal bovine serum (FBS Capricorn Scientific, Germany), penicillin (100 U/mL), streptomycin (100 mg/mL), and 25 μg/mL amphotericin B as an antimycotic. The disks and extraction solutions were incubated at 37°C in a humidified atmosphere of 5% CO_2_ for 24, 48, and 72 h.

Three disks were created for each of the nine concentration‐duration groups. After incubation, the culture medium containing material extracts was filtered through 0.22 µm membrane filters (Millipore, Sigma, St. Louis, MO, USA) to obtain sterile eluates for later cell application. The leached components were extracted according to ISO (ISO 10993‐5:2009) (Al‐Khatieeb, Mohammed, and Al‐Attar 2015; Eliades and Bourauel 2005; Finnema et al. 2010).

A rat embryo fibroblast cell line (REF, BSCL.138, IZSLER, Brescia, Italy) was grown in RAMPI 1640 with 10% FBS with penicillin (100 U/mL), streptomycin (100 mg/mL) and 25 μg/mL amphotericin B as an antimycotic. The monolayer cell culture was grown with prepared media in sterile polystyrene T‐75 flasks (Barloword Scientific Italia S.R.L., Milan, Italy) in a humidified incubator at 37°C and 5% CO_2_. The media was changed twice a week. The culture was monitored using a phase contrast inverted microscope (Leitz, Germany).

When cells reached 80% confluence (logarithmic growth phase), a mixture of 0.25% trypsin and 0.02% ethylenediaminetetraacetic acid (EDTA) was added for cell detachment and cell suspension. The detached cells were counted by mixing 10 μL of cell suspension with 10 μL of Trypan Blue vital dye, then using an automated cell counter (Thermo Fisher Scientific, MA, USA). All tests were performed between the seventh and ninth passages (Pagano et al. 2021; Kirby 2021).

### 2.4 MTT Assay

Based on the ISO (10993‐5:2009), the MTT assay (Intron Biotechnology, Korea) was applied to assess the viability of living cells in response to the extract of the four groups in the three concentration‐duration groups (24, 48, 72 h). The MTT test occurred in three separate experiments and in triplicate for each sample. The cell was seeded at a density of 10,000 cells per well in a 96‐well flat bottom plate. Each well was entirely filled with culture medium, then covered with parafilm, stirred gently, and stored for 24 h in a standard environment (37°C, 5% CO_2_, 95% humidity).

The culture medium was then substituted with the culture medium containing the concentration group extracts of the bonding material disks and an exposure time of 24 h. Measurements were conducted in triplicate. Four extract groups (each consisting of nine extracts) were subdivided into three subgroups based on immersion periods. Three of the nine wells belonged to a specific immersion period, and the negative control group included nine wells containing fresh culture media. When the exposure time elapsed, 20 uL of MTT solution was added to each well and incubated for 4 h. The yellow water‐soluble MTT solution forms a blue‐violet formazan product if viable cell metabolism occurs. Then, the media and MTT solution were replaced by dimethyl sulfoxide (100 μL per well) for 20 min. All procedures were conducted in a biological safety cabinet under sterile conditions (Dorminey, Dunn, and Taloumis 2003). The formazan concentration is reflected by color darkness; it was measured by optical density (OD) using a plate reader at 570 nm (Epoch, BioTek, Winooski, VT, USA).

The viability test was calculated according to the following formula:

$$\mathrm{Viab}.\%=100\times {\mathrm{OD}}_{570e}/{\mathrm{OD}}_{570b},$$

where viable % is the percentage of living cells, *OD*_570e_ and *OD*_570b_ represent the negative samples from the optical density of the test and the control, respectively (Lee et al. 2017).

### 2.5 DC

The four concentration groups were tested before and after polymerization using FTIR spectroscopy (Thermo Fisher Scientific, Waltham, MA, USA). The FTIR detected the ratio of the absorbance intensity of the aliphatic and aromatic functional groups c═c peak at 1637–1639 cm^−1^ and 1607–1609, respectively. The DC was calculated according to the following equation:

$$\mathrm{Dc}\%=1-(1638\;\;{\mathrm{cm}}^{-1}/1608\;\;\;{\mathrm{cm}}^{-1}\;\mathrm{cured}/1638\;\;\;{\mathrm{cm}}^{-1}/1608\;\;\;{\mathrm{cm}}^{-1}\;\mathrm{uncured})\times 100.$$

For non‐cured resin, each resin was placed directly on top of the device crystal for analysis. The cured resin included the preparation of five resin disks (*n* = 5) from each group (total of 20 disks). Preparation was the same as for preparing resin disks for the cytotoxicity test, except that a glass slide was used instead of a translucent polyethylene film to secure the flat surface of the disk, a primary condition for the FTIR analysis. After curing, the disks were subjected to FTIR spectroscopy analysis (Pagano et al. 2021).

### 2.6 SBS

A total of 176 human teeth were randomly divided into four concentration groups. Each tooth in each group (*n* = 44) was embedded in cold cure acrylic inside a PVC mold so that only the coronal part was exposed. Two notches were placed at the roots of the teeth to increase their retention inside the acrylic (Dorminey, Dunn, and Taloumis 2003; Proença et al. 2020) and fixed with soft wax (Quayle Dental Company, Worthing, England).

Using the surveyor (Paraline, Dentaurum, Pforzheim, Germany), all teeth were aligned, so the brackets were vertically aligned (Kavrik and Kucukyilmaz 2019; Proença et al. 2020). The G2BU adhesive was used in SE mode according to modified manufacturer instructions. This modification replaced the enamel etching/enamel bur cut procedure with polishing the tooth in a non‐fluoridated pumice/water slurry with a rubber cup for 15 s (Proença et al. 2020). P was subsequently applied using a disposable tip, left for 10 s, then dried for 5 s with maximum air pressure according to the manufacturer's instructions. For each of the four concentration groups, the previously prepared 2B was applied.

Each 2B concentration was applied with a disposable applicator over the enamel surface already treated with P. The 2B was then dried with gentle air to achieve a homogeneous thickness. It was then light‐cured for 10 s. Finally, a small amount of the adhesive paste (Transbond XT light cure adhesive past 3 M Unitek, Monrovia, USA) was applied on the base of the bracket with a surface area of 9.81 mm^2^ (Roth 0.022‐inch, DB. Co, England).

The bracket was bonded on the middle labial aspect of each tooth. A 200‐g standardized constant load was applied to the bracket for 10 s by the vertical arm of the surveyor during bracket cementation. Any excess adhesive was gently removed by the dental probe. For polymerization, a light curing device was adjusted to approximately 3 mm with an angle of 45° from the proximal surface of the bracket. The curing time was 20 and 10 s on each proximal side (Kavrik and Kucukyilmaz 2019; Dorminey, Dunn, and Taloumis 2003).

After the bonding procedure, each group was randomly subdivided into two subgroups (22 teeth per concentration group) according to the aging procedure. The first subgroups were incubated in distilled water at 37°C for 24 h. The other subgroups underwent 5000 thermocycles (Garma and Ibrahim 2023). Next, the SBS for the eight subgroups was tested using an Instron machine (Instron Laryee WDW‐50, Beijing, China). The chisel of the Instron machine acted on the base of the occlusal margin of the bracket and parallel to the bonded interface. Using a cross‐head speed of 0.5 mm/min, an occlusal‐gingival load was applied until the bracket debonded. The results were calculated by dividing the load in Newtons by the surface area of the bracket base (1 MPa = 1 N/mm^2^).

### 2.7 Statistical Analysis

The statistical analysis was performed using SPSS (Version 25 SPSS Inc., Chicago, USA). All data were first checked for normality using the Shapiro–Wilk test and for homogeneity of variances using Levene's test. Once the assumptions for normality and homogeneity were confirmed, descriptive statistics were calculated, including means and standard deviations for all measured parameters.

For inferential statistics, one‐way analysis of variance (ANOVA) was used to compare the differences between the groups. When the ANOVA indicated significant differences, post‐hoc comparisons were performed using Tukey's Honestly Significant Difference (HSD) test to identify which specific groups differed from each other. The significance level was set at *p* < 0.05 for all statistical tests. For evaluating cytotoxicity, the MTT assay data were analyzed using ANOVA to assess cell viability differences among the groups at various immersion periods (24, 48, and 72 h). Significant differences were identified and further analyzed using Tukey's HSD test. ANOVA was also applied to the DC data, comparing the different concentrations of TCSNp. The statistical analysis in this study employed both ANOVA for initial comparisons and Tukey's HSD for detailed post‐hoc analysis to determine specific group differences. The results were considered significant at *p* < 0.05, ensuring rigorous statistical evaluation of the data.

## 3 Results

### 3.1 Cytotoxicity Tests

Cell viability increased with higher concentrations of TCSNp. After 72 h, significant differences in cell viability were observed between the control and 5% TCSNp groups (*p* = 0.014) as shown in Figure 1 and Table 1.

Figure 1Cell viability in different groups. Control negative control cells. Control cells treated with 2B after 24 h. Control cells treated with 2B after 48 h. Control cells treated with 2B after 72 h. Cells treated with 1% 2B after 24 h. Cells treated with 1% 2B after 48 h. Cells treated with 1% 2B after 72 h. Cells treated with 3% 2B after 24 h. Cells treated with 3% 2B after 48 h. Cells treated with 3% 2B after 72 h. Cells treated with 5% 2B after 24 h. Cells treated with 5% 2B after 48 h. Cells were treated with 5% 2B after 72 h.

**Figure:**
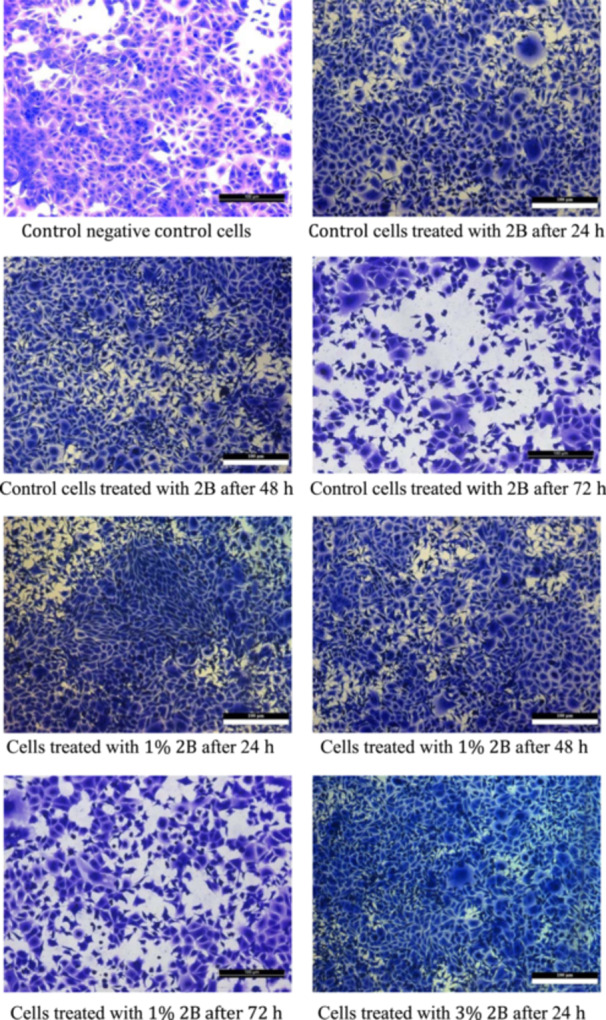

**Figure:**
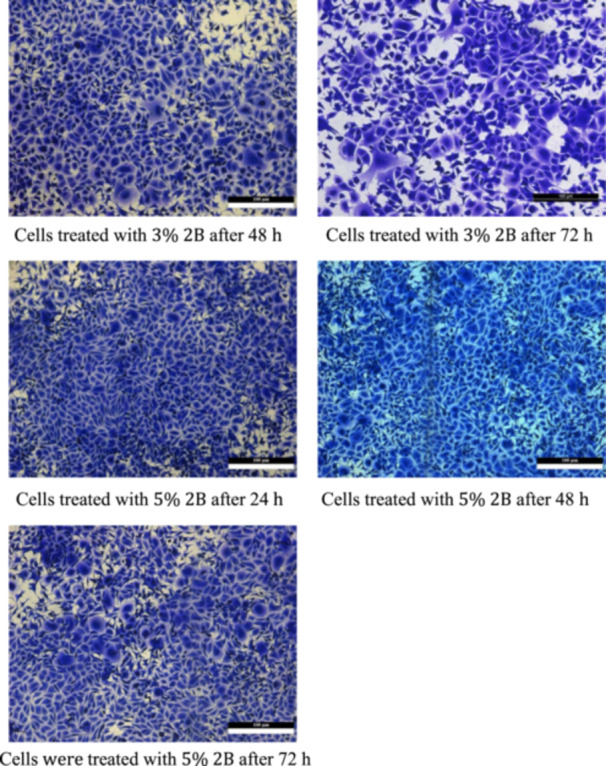

**Table 1:** Descriptive statistics of the percentages of cell viability in different groups.

Group	Immersion period (h)	*N*	Mean	SD	Min.	Max.
Control 2B	24	3	86.66	2.081	85	89
	48	3	82.66	1.52	81	84
	72	3	79.66	1.52	78	81
1%2B	24	3	88.66	1.52	87	90
	48	3	85	3	82	88
	72	3	81	1	80	82
3%2B	24	3	89.66	1.52	88	91
	48	3	85.33	1.52	84	87
	72	3	81.33	1.52	80	83
5%2B	24	3	91	0.57	90	92
	48	3	87.66	0.33	87	88
	72	3	84	1	83	85

**Figure 2:**
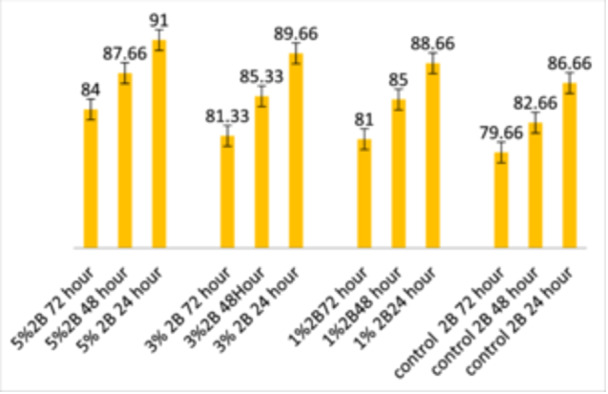
Rat embryo fibroblast cell lines exposed or not exposed to extracts of 2B and treated with different concentrations of TCSNp and stained with crystal violet.

Table 1 shows the descriptive statistics of the cytotoxicity test by concentration group and duration. Table 1 shows cell viability percentages in different groups, segmented by immersion periods (24, 48, and 72 h) and concentration groups (Control, 1%, 3%, 5% 2B). The maximum cell viability in 24 h of water storage was in group 5%2B (91% ± 0.57). The minimum cell viability in 24 h was the control group (85% ± 2.08). There are similar results for the 48 and 72 h durations. Figure 2 shows the cell viability in different with varying concentrations. This suggests that cell viability may increase with higher concentrations of TCSNp.

Table 2 showcases the ANOVA test results for the cytotoxic effect based on immersion time across all groups, providing *F*‐test and *p*‐values for immersion periods of 24, 48, and 72 h. The ANOVA test in Table 2 found no significant differences in cell viability for the 24 or 48‐h immersion periods. There was a significant difference for the 72‐h immersion period between the control and the 5%2B group. In pairwise comparisons between immersion periods (Table 3), cell viability was significantly different between the 24 h and both 48 and 72 h groups. However, there were no significant differences between the durations of 48 and 72 h.

**Table 2:** ANOVA test for the cytotoxic effect based on immersion time across all groups.

Immersion period (h)	*F*‐test	*p*‐value
24	4	0.052
48	3.58	0.066
72	5.93	0.020
Tukey HSD test between 2B and 5%2B group		0.014

**Table 3:** ANOVA test for the cytotoxicity effect between immersion periods.

Variable	*F* value	*p*‐value
ANOVA	15.258	0.001

Tukey HSD test	*p*‐value	
24 h vs. 48 h	0.047	
24 h vs. 72 h	0.001	
48 h vs. 72 h	0.058	

### 3.2 DC

**Figure 3:**
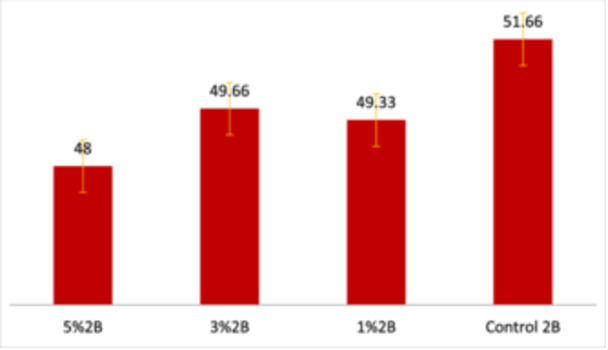
Degree of conversion among the different concentration groups.

The mean DC values decreased slightly with higher concentrations of TCSNp as shown in Figure 3. ANOVA indicated no significant differences between groups as shown in Table 4.

**Table 4:** Descriptive statistics and ANOVA test for the degree of conversion among concentration groups.

Groups	Descriptive statistics					Comparison	
	*N*	Mean	SD	Min	Max	*F*‐test	*p*‐value
Control	3	51.66	1.52	50	53	2.760	0.112
1%2B	3	49.33	2.08	47	51		
3%2B	3	49.66	1.52	48	51		
5%2B	3	48	1	47	49		

Table 4 shows the descriptive statistics of DC. The highest mean DC in the control group was 51.66 ± 1.52. It dropped incrementally as the percentage of TCSNp increased to reach a minimum value was 48 ± 1 in group 5%2B. There were no significant differences between the groups as shown in Figure 3.

### 3.3 SBS

**Figure 4:**
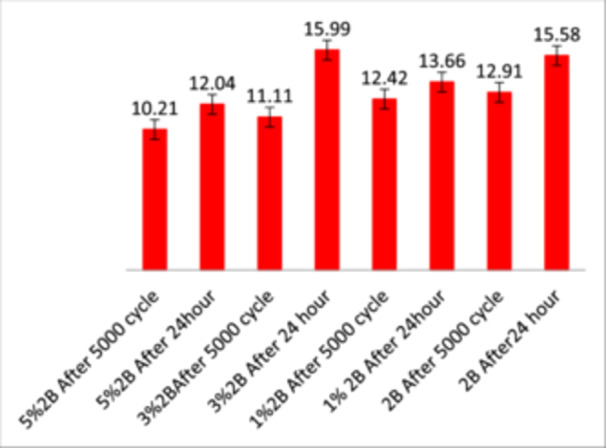
Descriptive and comparative values for the shear bond strength measured in MPa for all concentration groups after aging (24‐h water storage and 5000 cycles).

ANOVA showed significant differences between groups (*p* < 0.05), except between the control and 3% TCSNp groups as shown in Figure 4. Post‐hoc Tukey's HSD tests identified significant differences between the control and both 1% and 5% TCSNp groups as shown in Table 5.

**Table 5:** Descriptive and comparative statistics for the shear bond strength of all concentration groups after aging.

Groups	Aging	*N*	Mean	SD	Min.	Max.	*p*‐value
Control 2B	24 h	22	15.58	1.5	12	18	0.000
	5000 cycles	22	12.91	1.55	9.9	14	
1%2B	24 h	22	13.66	2.34	10	18	0.041
	5000 cycles	22	12.42	1.41	10.2	15	
3%2B	24 h	22	15.99	1.81	13.5	19	0.000
	5000 cycles	22	11.11	1.42	9	14	
5%2B	24 h	22	12.04	2.32	8.2	16	0.000
	5000 cycles	22	10.21	1.81	8.1	13	

Table 5 shows the highest mean SBS was 15.58 SD 1.1 MPa after 24 h of water storage and 12.91 ± 1.55 MPa after 5000 thermocycles. Figure 4 displays descriptive and comparative statistics for the SBS of all concentration groups after aging (24‐h water storage and 5000 cycles).

Table 6 shows post‐hoc tests for the 24 h aging condition found significant differences in SBS between the control and both 1%2B and 5%2B, but no difference between the control and 3%2B.

**Table 6:** ANOVA test for shear bond strength between all groups after 24 water storage and 5000 thermocycles.

Type of aging	*F* value	*p*‐value
24 h ANOVA test	17.79	0.000*
	Tukey HSD	
2B	1%2B	0.012*
	3%2B	0.909
	5%2B	0.000*
5000 thermocycle ANOVA test	*F* value 15.081	0.000*
	Tukey HSD	*p*‐value
2B	1%2B	0.693
	3%2B	0.001*
	5%2B	0.000*

* Significant at *p* < 0.05.

For the 5000 thermocycle condition, there were significant differences between the control and both 3%2B and 5%2B. The 1%2B group was not significantly different from the control. An independent *t*‐test shows a significant difference in the SBS value between both aging procedures (Table 7). This means that the SBS was significantly reduced after the aging procedure with 5000 cycles.

**Table 7:** Independent samples *t*‐test for the shear bond strength value of all groups between 24 water storage and 5000 thermocycles.

	Levene's test		*t*‐test	
Groups	*F* value	Sig.	*df*	Sig (2‐tailed)
2B	0.263	0.611	42	0.000*
1%2B	5.732	0.021	42	0.041*
3%2B	1.578	0.216	42	0.000*
5%2B	0.46	0.830	42	0.000*

* *Significant at *p* < 0.05.

## 4 Discussion

In this study, we investigated the SBS, cytotoxicity, and degree of universal conversion (DC) of G2 bond (G2BU) when modified with TCSNp. Overall, the findings indicate that the G2BU adhesive system maintains clinically acceptable SBS levels even when altered with TCSNp.

The original protocol provided by the G2BU manufacturer was modified, substituting the enamel bur cut with an enamel surface polish. This modification was designed to achieve a bond strength lower than the 46 MPa observed by the researchers after 24 h. In the context of orthodontic adhesives, reduced bond strength is not necessarily detrimental; it preserves enamel integrity and minimizes potential damage during debonding at the end of treatment.

The rationale behind adding TCSNp to G2BU adhesive as a filler material may provide an alkaline environment, calcium ion release and hydroxyapatite formation (Zhao and Chang 2004; Tan et al. 2020). Consequently, this approach may help maintain enamel health and reduce demineralization. All these merits explain why TCSNp was added to adhesive 2B as a filler material to obtain some or all of the benefits of TCSPN. TCSNp has self‐setting behavior within a moist environment; it forms calcium silicate hydrate (C‐S‐H gels) and calcium hydroxide (Tan et al. 2020), which could interfere with the acid monomer of the P. The rationale for adding TCSNp to 2B was to leave the functional monomer in P (particularly 10 MDP and 4‐MET) to act properly on the enamel surface (away from the basic media of TCSNp). The TCSNp was suspended in 2B resin only (not to the hydrophilic P) but would not interact chemically with 2B. Since TCSNp was suspended in the second step, it could act as a basic sandwich media between the surface of the tooth and adhesive paste (Zhao and Chang 2004; Strassler and Levin 2012; Tan et al. 2020).

### 4.1 SBS

Although there is no consensus on the ideal bond strength for orthodontic applications, researchers generally consider a range of 5.9–7.8 MPa to be the minimum acceptable bond strength for bracket bonding that still yields satisfactory clinical outcomes (Reynolds 1975; Al‐Khatieeb, Mohammed, and Al‐Attar 2015). Most in vitro studies found a bond strength between 6 and 12 MPa acceptable (Eliades and Bourauel 2005; Finnema et al. 2010). This minimum SBS in all groups was 10.21 MPa in the 5%2B group after the 5000 thermocycles, and the maximum value was 15.99 MPa in 3%2B after 24 h of water storage (Figure 4, Table 5). This result is consistent with other authors (Finnema et al. 2010; Al Azzawi et al. 2021). As the concentration of TCSNp increased, there was a corresponding decrease in SBS. Although the reduced SBS remained within clinically acceptable limits, this decline could be correlated with a decrease in the DC. This reduction in DC might result in a slower polymerization rate, which could adversely affect the mechanical properties of the material (Al‐Sarkhi and Al‐Groosh 2017). There were significant differences in SBS between some of the groups tested with both aging procedures (Table 6). This indicates that TCSNp can affect the SBS value. However, SBS remained within clinically acceptable parameters as shown in Table 7, suggesting that a G2BU adhesive incorporating TCSNp could be an appropriate option for use as an orthodontic adhesive in SE mode.

The significant difference in the SBS value in all tested groups between 24‐h water storage and 5000 thermocycles (Figure 4, Table 7) suggests that the G2BU adhesive does not resist deterioration with aging procedures. This finding is in disagreement with Iwase et al (Iwase et al. 2022)., who suggested that G2BU is less sensitive to changes in conditions than other adhesives. This could be attributed to the addition of the TCSNp and the modification of the adhesive system from bur cut to enamel polishing. Interestingly, ANOVA revealed significant variations in SBS between the different TCSNp concentration groups of TCSNp, corroborating studies that have demonstrated the impact of additive concentrations on bond strength (Camilleri, Laurent, and About 2014).

### 4.2 Cytotoxicity Tests

Cytotoxicity tests are crucial when preparing or adding new material to existing substances. Moreover, these findings help to confirm that any resultant reactions are nontoxic to living tissues, particularly if the material comes in contact with the gingiva and/or oral mucosa in an accidental manner. TCSNp is a proven bioactive and biomimetic material. TCSNp has the ability to stimulate cell proliferation and form a chemical bond with bone. This may explain why the cell viability test increased with an increase in the concentration of the TCSNp. However, this was only significant between the control and 5%2B groups for immersion (Tables 1 and 2) (Tan et al. 2020; Bossù et al. 2021; Song et al. 2021). There are other possible causes of the increase in cell viability with TCSNp. The reaction of the TCSNp to a humid environment leads to the development of C‐S‐H gel and Ca (OH)_2_ (Tan et al. 2020). These two products may interact with the free, unreacted monomer or the self‐setting properties of the TCSNp; this would block the release of the unreacted free monomer by decreasing the permeability of the material (Figure 4) (Singh 2015).

The cytotoxicity results are of significant interest, particularly the immersion time for the comparison between groups 2B and 5%2B. Previous studies have raised concerns about the biocompatibility of dental materials (Murray, García Godoy, and García Godoy 2007), making our findings a valuable addition to the existing literature on the safety of orthodontic adhesives.

Cell viability was found to be only significantly reduced between the 24 h condition and the 48 and 72 h immersion. It did not differ between 48 and 72 h (Figure 1, Table 3). This may indicate that cell viability is dose‐dependent in the first 48 h of immersion (Figure 1). Nevertheless, all findings of this study concerning cell viability were far above the 70% level required by international standards (10993‐5:2009), which suggests that the experimental groups have acceptable cytocompatibility (Dorminey, Dunn, and Taloumis 2003). It is essential to evaluate cytocompatibility before the intraoral use of newly developed adhesives.

### 4.3 DC

The DC is the ratio of how many monomers are converted into polymers (polymerization ratio). DC is related to the physical and mechanical properties of the polymer. FTIR spectroscopy is the most common and reliable method for measuring the DC (Mohammed and Riad 2019; Bin Nooh et al. 2020). As the concentration of TCSNp increased, DC decreased, but not significantly (Table 4). This decrease in DC can be explained by an increase in adhesive viscosity following the addition of TCSNp. Increased viscosity would adversely affect DC (Kirby 2021). The maximum value of DC was 51.66 in the 2B group, and the minimum was 48 in the 5%2B group as shown in Figure 3. These are apparently low values. Studies using other types of SE adhesives have found similar or even lower values. This may be attributed to the cooling effect of polymerization, an exothermic reaction in which an elevation in temperature leads to more polymerization. The room might absorb the heat of this vitro test if the room temperature of 20°C and body temperature of 37°C were compared (Mohammed and Riad 2019). However, the correlation between DC and mechanical properties, such as SBS, should be a focus of future research.

The study also brought to light the impact of modifying the manufacturer's instructions. The substitution of the enamel bur cut with pumice polishing could have significant clinical implications, particularly in the preservation of enamel integrity during debonding.

This study is not without limitations. The SBS was assessed after only 24 h and 5000 thermocycles. Longer‐term studies could provide more insight into the stability and longevity of the bonds formed. The study found no significant differences in DC across groups, which leaves room for further investigation into how DC could impact SBS and cytotoxicity in the long term. Cytotoxicity was examined at only one time point (72 h), which may not fully represent the biological implications of using TCSNp in clinical settings. The study implemented a modification of the manufacturer's protocol by replacing the enamel bur cut with pumice polishing, which is a deviation that may not be applicable in all clinical situations. The study used only three different concentrations of TCSNp (1%, 3%, and 5%). Evaluating a broader range of concentrations could provide a more comprehensive understanding.

It would be beneficial to compare the modified G2BU adhesive system with other existing orthodontic adhesive systems to assess its relative efficacy and safety. To confirm the laboratory findings, clinical trials should be carried out to investigate the performance in real‐world scenarios, which could include factors such as oral hygiene and diet. Given that the study suggested that the rate of polymerization could affect mechanical properties, future research should explore the mechanical properties of the adhesive system in more detail. Further research should be conducted to evaluate the long‐term effects of modifications to the G2BU adhesive system, especially in terms of SBS, cytotoxicity, and DC.

## 5 Conclusion

The study investigated the effects of adding TCSNp to the G2BU in SE mode, focusing on SBS, cytotoxicity, and DC. The addition of TCSNp to G2BU in SE mode maintained clinically acceptable SBS levels. After 24 h of water storage, the highest SBS was observed in the 3% TCSNp group, while the lowest was in the 5% TCSNp group. After 5000 thermocycles, SBS values decreased across all groups but remained within acceptable limits. The significant differences in SBS between some groups suggest that TCSNp concentration can affect bond strength.

The cytotoxicity results indicated that cell viability increased with higher concentrations of TCSNp, with significant differences observed between the control and 5% TCSNp groups after 72 h. All tested concentrations maintained cell viability above the 70% threshold required by international standards, indicating acceptable cytocompatibility.

The DC slightly decreased with higher concentrations of TCSNp, but ANOVA indicated no significant differences between groups. The highest DC was observed in the control group, while the lowest was in the 5% TCSNp group.

In conclusion, adding TCSNp to the G2BU adhesive in SE mode can maintain clinically acceptable SBS levels and enhance cytocompatibility, though higher concentrations may slightly reduce SBS and DC. The findings support the potential clinical utility of G2BU modified with TCSNp. However, further comprehensive studies are needed to confirm these results and evaluate the long‐term effects of TCSNp incorporation, particularly regarding SBS, cytotoxicity, and DC.

## Author Contributions

Y.R.A. and M.A. conceived the idea of study, experimental design, and data collection. S.K. and S.J.A.Z. performed data pretesting and statistical analysis. S.J.A.Z. copyedited and proofread the manuscript. All authors reviewed the paper, gave final approval, and agreed to be accountable for all aspects of the work.

## Ethics Statement

The study received ethical approval from the Ethics Committee of the College of Dentistry, University of Baghdad (Ref. number: 693) dated 10/11/2022. This was an in vitro study, and no humans were involved in this study. All experiments were performed in accordance with relevant guidelines and regulations (such as the Declaration of Helsinki).

## Consent

The authors have nothing to report.

## Conflicts of Interest

The authors declare no conflicts of interest.

## Data Availability

The data that support the findings of this study are available from the corresponding author upon reasonable request.
